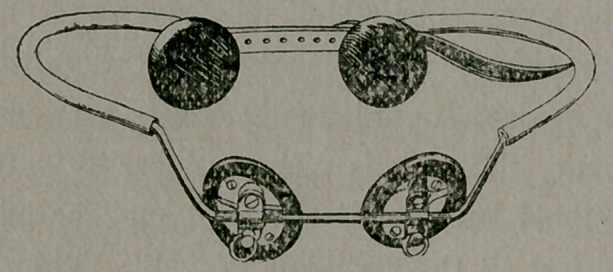# Some Personal Observations in the Fitting of Hernia-Supports

**Published:** 1907-05

**Authors:** Arnold H. Lindorme

**Affiliations:** Atlanta, Ga.


### SOME PERSONAL OBSERVATIONS IN THE FITTING OF HERNIA-SUPPORTS.*

By ARNOLD H. LINDORME, M.D., Atlanta, Ga.

The treatment of hernia, other than operative, is as old, at least, as the Egyptian mummies: it is said that braces were found on mummies in Egypt, which, it was claimed, were trusses for the support of ruptures.

I wish to bring out for your consideration some points which may prove of interest to you.

Supporting a rupture with a truss is only a palliative treatment, not a cure, except in rare cases of children; and a great many persons wearing trusses would be better off if they would go without one, instead of continuing to wear what they have. I claim that not all cases of hernia are the proper cases to wear trusses. Indirect hernia is the easiest to fit with a truss properly in the beginning of hernia, for the breach is far enough above the pubic bone, so that the truss-pad, being placed over the ring, will not impinge on the same. I care not how soft the pad; if it presses hard enopgh to hold the rupture back, it will give pain in proportion to the amount of pressure exerted. Ruptures which have never been supported properly, always allowing a part of the hernial sack to protrude, will form adhesions, and adhesions, once formed, will under the

*Read before the Fulton County Medical Society, February 21, 1907.

pressure of the truss always be made worse. If the bowel, or omentum, is returned into the abdomen, and, as result of the adhesions formed, the hernial sack will remain in the scrotum, the truss-pad, when placed over the ring, will press the sides of the sack together, keeping back a portion of the bowel or omentum, not conspicuous except to the very inexperienced observer. But it will require so much pressure to accomplish this, as to make the wearing of a truss not only painful, but dangerous.

More care should be exercised in the proper fitting of braces. A drug-clerk is not the fit person to fit a brace. I have had cases of varicocele, hydrocele and enlarged lymphatic gland sent to me to have a truss applied.

A hernia is a surgical disease always. The doctor who sends his case of hernia to the druggist does not do it justice. Incurable cases and such as are very troublesome operating cases are made by faulty appliances. Examination and the fitting of a truss require as much anatomical knowledge as does the radical operation.

A person applying to me for a truss is by me always carefully examined before taking his measurement, even, for I have found that errors in diagnosis are with difficulty avoided. The history of the case will sometimes be very plain as to the onset of the rupture, then again the patient will not know when or how the trouble appeared first. In this connection it is important to know how long the patient has been ruptured, if he has put the truss on himself, and if it has always held the rupture well up; if not, then I expect to find adhesions.

In having the patient stand up before you, let him cough; if there is an impulse, it will be a point in favor of rupture. Then again a hydtocele may be so large as to extend to the external ring, and assume the appearance of a large hernia. With the light-test and the hypodermic needle all will be cleared up.

Speaksng of hydrocele in children, it is apropos to add that they will deceive the doctor often, an opening existing through which fluid will pass in and out of the abdomen.

Varicocele, enlarged lymphatic gland, can usually in this region be differentiated; but I have seen a few cases where enlarged lymphatic glands and rupture existed together, a condition which makes it a hard case for a truss.

Having gleaned ali that I can learn of the patient in the upright position, I next examine him in the recumbent posture; by reducing the rupture something may be learned. First and most important: Does all of the rupture return into the abdominal cavity? (by all of the rupture is meant the hernial sack, as well as its contents.) By invaginating the scrotum with the finger, pressing up until the point of the finger gets within the ring, you can make out if all of the hernia has been returned. If the pillars of the ring can be plainly made out, the chances are, your rupture is in; but if there seems to be something which rolls over the end of the finger, the difficulty in making out the ring—in other words, if there is a curtain, so to speak, which continually comes between the examining finger and the ring, the sack is either adherent around the edge of the ring and doubled back on itself, or it may be adherent not only around the ring, but in the scrotum as well. Then, if a trus-pad is placed on such a rupture, the sack is pressed together, giving pain and increasing the adhesion and subsequent trouble. A rupture which is not adherent and which, with its sack, has been entirely returned, even if it is large and descends into the scrotum, can, with a properly fitted truss, be supported with safety.

If we find that the pelvic bone makes the floor of the opening, the result is, as I said before, a difficult case, as even a soft pad, pressing on the bone, gives pain. This difficulty is the surest indication in point to properly close in the radical operation for the cure of hernia.

The measurement having been carefully made, the truss-frame is then constructed thereby. But the most difficult task is yet before us: it is the fitting of the spring, which the truss-frame practically is, to conform to the body of the patient. The patient must have free movement of his body, the pressure of the pad over the ring being sufficient to retain the hernia under the strain of physical exertion of his daily labor. But above all, it must be comfortable. I have a truss here with me which I will explain to you presently.

I have made an interesting observation in fitting corpulent patients with a support for umbilical hernia. The thick layer of fat over the abdominal muscles will prevent the truss-pad doing its proper work, unless specially constructed to meet the difficulty. I have a photo of it here with me.

I have fitted up about 500 cases. Of that number, six were women, ten babies, and thirty-two children, of whom three were girls.

I know of a few cases of babies, which I have been able to keep up with, who were cured by the support, inguinal and umbilical hernia.

I show the style of truss I have devised, and use in fitting inguinal hernia. It is made of a suitable spring-wire, and so shaped as to fit accurately the body. The front part carrying the pads is made so as to reach from one groin to the other. The short up-right part is bent so as to be the same angle as the wearer's groin. The side-curves, which reach around to the back-pads, are so bent as to fit around the body, above the hip-joint, and below the crest of the ilium. And the whole can be so shaped as to give as much pressure as is necessary to retain the parts in place, or as little as may be indicated in the special case in hand.

The pads used in front are of two kinds, either hard wood, or soft water-pads. And they can be adjusted on the frame, allowing thereby of a very accurate adjustment.

The back-pads rest on either side of the spinal column. They are made of hard-rubber.

102 1-2 Whitehall St.

## Figures and Tables

**Figure f1:**